# Design and Implementation of a Modular UUV Simulation Platform

**DOI:** 10.3390/s22208043

**Published:** 2022-10-21

**Authors:** Zekai Zhang, Weishi Mi, Jun Du, Ziyuan Wang, Wei Wei, Yuang Zhang, Yutong Yang, Yong Ren

**Affiliations:** 1 Tsinghua Shenzhen International Graduate School, Tsinghua University, Shenzhen 518055, China; 2 Department of Electronic Engineering, Tsinghua University, Beijing 100084, China; 3 Department of Automation, Tsinghua University, Beijing 100084, China

**Keywords:** unmanned underwater vehicle (UUV), self-feedback development framework, modular simulation platform, robotic operating system (ROS), multi-UUV formation control

## Abstract

The complex and time-varying marine environment puts forward demanding requirements for the structural design and algorithm development of unmanned underwater vehicles (UUVs). It is inevitable to repeatedly evaluate the feasibility of autonomy schemes to enhance the intelligence and security of the UUV before putting it into use. Considering the high cost of the UUV hardware platform and the high risk of underwater experiments, this study aims to evaluate and optimize autonomy schemes in the manner of software-in-loop (SIL) simulation efficiently. Therefore, a self-feedback development framework is proposed and a multi-interface, programmable modular simulation platform for UUV based on a robotic operating system (ROS) is designed. The platform integrates the 3D marine environment, UUV models, sensor plugins, motion control plugins in a modular manner, and reserves programming interfaces for users to test various algorithms. Subsequently, we demonstrate the simulation details with cases, such as single UUV path planning, task scheduling, and multi-UUV formation control, and construct underwater experiments to confirm the feasibility of the simulation platform. Finally, the extensibility of the simulation platform and the related performance analysis are discussed.

## 1. Introduction

With the booming development of the “blue industry”, traditional marine operation methods can no longer keep up with the demand. Thanks to its intelligence and mobility, unmanned underwater vehicles (UUVs) have been widely used in environmental observation, resource exploration, biological survey, disaster prediction, auxiliary positioning, and other underwater tasks [1,2,3]. What is more, UUV-assisted marine information networks and related variants are emerging in an endless stream, such as the maritime giant cellular network [4] and age of information (AoI) [5] inspired underwater information networks [6,7]. Unfortunately, the variable current environment leads to poor robustness of UUVs in environmental perception, motion control, cluster collaboration, etc., which greatly hinders the rapid advancement of marine science [8].

Path planning and formation control are two core concepts in UUVs [9]. On the one hand, as the most basic and critical part of UUVs, path planning directly determines the accuracy and efficiency of the UUV’s mission execution. The challenge of path planning is to plan a collision-free, smooth, shortest path from the starting point to the target point, while considering the unknown environment and its own energy consumption limitations at the same time [10]. Recently, techniques, such as artificial potential field, deep neural networks, and reinforcement learning, have been introduced into the path planning of unmanned underwater vehicles with good results [11,12,13,14,15]. On the other hand, with the diversification and complexity of underwater observation missions, UUV group cooperation has become an inevitable trend, and formation control has gradually become a challenging topic. The representative methods for UUV formation control include virtual structure, behavior structure, artificial potential field, neural networks, etc. [16,17,18,19,20]. Previously, path-planning methods and formation control strategies applied in UUVs were mainly migrated from ground unmanned vehicles and low-altitude unmanned aerial vehicles (UAVs), and it is difficult to ensure the success rate and efficiency of UUVs in the process of mission execution and may even lead to safety hazards due to the lack of analysis of the time-varying underwater environment. Thus, the autonomy schemes must be subjected to repeated underwater experiments before application.

Considering the high cost of hardware platform and the high risk of physical tests, robot simulation technology has gradually become a feasible solution for algorithm verification [21]. For example, Ojeda et al. [22] proposed a simulation framework based on GADEN and Unity Engine to integrate simulated olfaction and simulated vision into multi-sensor mobile robots. In [23], the authors developed a simulator to support the navigation activities of unmanned ground vehicles (UGV) based on ROS and Gazebo. For UAV autopilot systems, Dai et al. [24] developed RFlySim, an FPGA-based simulation platform using hardware-in-loop simulation. Xiao et al. [25] proposed a customizable multi-rotor UAV simulation platform based on ROS, Gazebo, and PX4 called XTDrone, which integrates visualized 3D scenes and sensor models and supports users to test algorithms. Chen et al. [26] designed an end-to-end UAV simulation platform for SLAM, navigation research, and applications, including detailed simulator setup, out-of-the-box positioning, mapping, and navigation systems. In [27], based on ROS and Gazebo, the authors proposed an integrated vision system for unmanned aerial vehicles landing on the targeted moving UGV platform and tested target detection and tracking strategies on it. However, the simulation simulators introduced above are mainly designed for air-based or land-based robots, while the field of underwater robots lacks high-quality simulators to support scientific research. The most representative is the UUV simulator introduced in [28], which is an extension of Gazebo for underwater scenarios and integrates related plugins to simulate underwater hydrostatic and hydrodynamic effects, thrusters, sensors, and external disturbances; the simulator is mainly used to simulate multiple underwater vehicles and intervention tasks using robotic manipulators. Based on the simulation architecture of the UUV simulator, the simulation of hybrid autonomous underwater vehicle was realized in [29], and the control strategy based on PID was verified. Nie et al. [30] adopted the strategy of combining fluid mechanics software and Unity3D to assist in the construction of a virtual ocean environment to simulate the working state and process of the UUV under different conditions; this simulation platform will be abbreviated as MVSPU in the following. In [31], a simulation platform for an intervention autonomous underwater vehicle (I-AUV) was proposed, characterized by providing advanced fluid dynamics based on actual geometry, a simulation of underwater sensors and actuators, and a realistic rendering of the underwater environment and ocean surface. In addition, the UUV simulation platforms implemented by hardware-in-loop simulation also have considerable application prospects [32,33].

The main indicators to evaluate the simulation platform are simulation accuracy, development difficulty, and practicability. However, most of the existing UUV simulation platforms have poor universality due to low accuracy of environment modeling, difficulty in secondary development, and transitioning to hardware platforms [28,29,30,31,32,33]. To overcome the above shortcomings, this study designs and implements a modular UUV simulation platform to test the autonomy schemes of UUVs. The main highlights are as follows:

(1). An efficient self-feedback development framework is proposed, and the role of the simulation platform in it is introduced.
(2). The simulation platform, including high-precision simulation scenarios, multi-source sensors, control plugins, and UUV models, is made in a modular manner.
(3). In this work, the robustness of the programming interfaces and the reliability of the simulation platform have been verified by constructing simulation tasks and underwater experiments.

The rest of this article is organized as follows. In Section 2, the self-feedback development framework and the implementation of the simulation platform are introduced. In Section 3, the co-simulation based on ROS and Matlab is presented. In Section 4, the proposed formation control strategy is verified in the simulation platform, and underwater experiments are conducted for comparison. In Section 5, the extensibility and superiority of the simulation platform are discussed. Finally, the conclusions and future work are given in Section 6.

## 2. Framework Design and Platform Implementation

### 2.1. Self-Feedback Development Framework for UUV 

**Figure 1:**
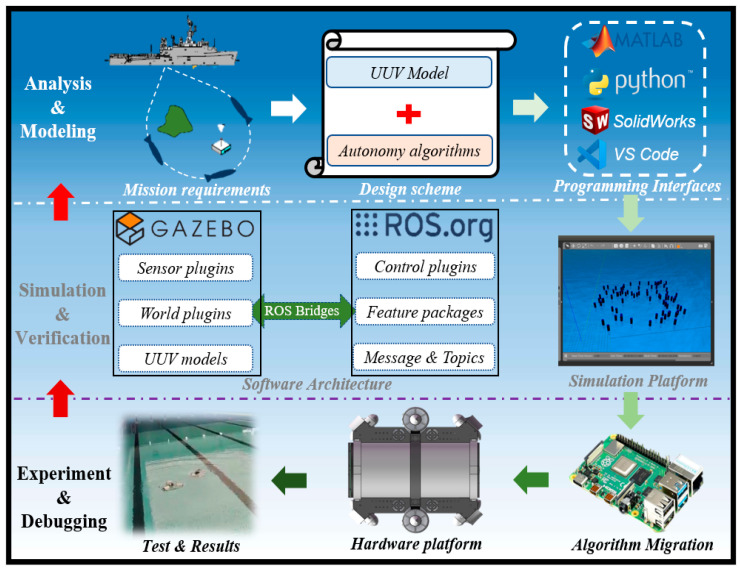
Illustration of the self-feedback development framework.

Thanks to the role of the simulation platform, the self-feedback development framework in Figure 1 organically links theory, simulation, and the experiment together, which improves the development efficiency of the UUV and avoids unexpected risks to a great extent. The recommended development process of this framework and software architecture of the simulation platform are introduced as follows:

- **Development Process:** The outer arrows in Figure 1 form a closed-loop development line where the researchers can design schemes according to task requirements, including UUV models (structure, controller, etc.) and autonomous algorithms (path planning, formation control, etc.), which are imported into the simulation platform for verification and optimization through relevant programming interfaces (Matlab, Python, VScode, Solidworks, Tsinghua University, Beijing). After obtaining satisfactory simulation results, the verified algorithms will be transferred to the control unit of the hardware platform to conduct experiments. In fact, simulation is not a substitution for experiments. If the experimental results are still unsatisfactory, debugging and even redesigning should be carried out by stepwise upward feedback.
- **Software Architecture:** According to the concept of modular design, the software packages of the simulation platform can be divided into two groups based on Gazebo and ROS, which both support secondary development. In Gazebo, UUV models, sensor plugins, and world plugins are stored to support simulation. In ROS, users can control and communicate with entities in Gazebo through control plugins and feature packages. It is worth mentioning that ROS is connected to Gazebo through ROS Bridges; thus, the related programming interfaces can subscribe messages and publish commands through corresponding topics.

### 2.2. Construction of Virtual Ocean Environment

The fidelity of the virtual ocean environment directly affects the accuracy of the simulation, and the key is to accurately depict the seabed, whether it is to support bathymetry missions or just to make the scene look more realistic. With several realistic Gazebo worlds already available in the UUV simulator, this work chooses the Ocean Waves World [28] with wave shaders for secondary development, focusing on accurate modeling of the seabed based on real ocean environment data.

**Figure 2:**
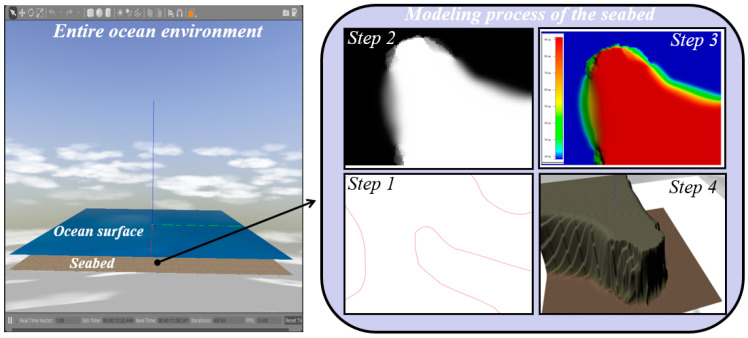
Visualization of the entire ocean environment and the modeling process of the seabed.

In our work, the commonly used S-57 file is selected as the main data source of the marine environmental data, and the seabed is modeled according to the real data provided. The S-57 file with a 0.000 extension is a type of electronic navigational chart (ENC) standardized by the International Hydrographic Organisation (IHO) that contains vector format data based on the S-57 object model [34]. It contains navigational information, such as sea depth, soundings, contours, and other information. All this data is available inside the file in a vector format and is totally independent of how it is displayed by S-57 readers. The S-57 files can be opened using applications, such as ESRI ArcGIS, OpenCPN, and APIs, such as GDAL. The modeling process of the seabed based on the hydrological data in the S-57 file is as follows: firstly, the Anaconda ogr2ogr library is used to view the hierarchical information of the S-57 file (0.000 file) and carry out non-visual processing operations, including format transformation. Then, the vector data in the S-57 file is transformed into raster data by Quantum GIS (QGIS), and the terrain file (.tif file) is obtained by interpolation of blank areas. After that, the Global Mapper software is used to convert it into a digital elevation model file (.dem file). Finally, the Python3-gdal library is used in the ROS-Gazebo environment to convert the dem file into the world file and display it in the Gazebo simulation environment; the corresponding visualization process is shown in steps 1 to 4 in Figure 2. In addition, QGIS is an open-source GIS desktop software that provides basic functionality and add-ons (Python or C++, Tsinghua University, Beijing) that allow users to browse, manage, edit, and analyze data and maps [35,36].

### 2.3. Design of 3D Model, Sensor Plugin, and Control Plugin for UUV

After the construction of the virtual ocean environment, it is necessary to create a UUV simulation entity with sensors and the control plugin.

**Figure 3:**
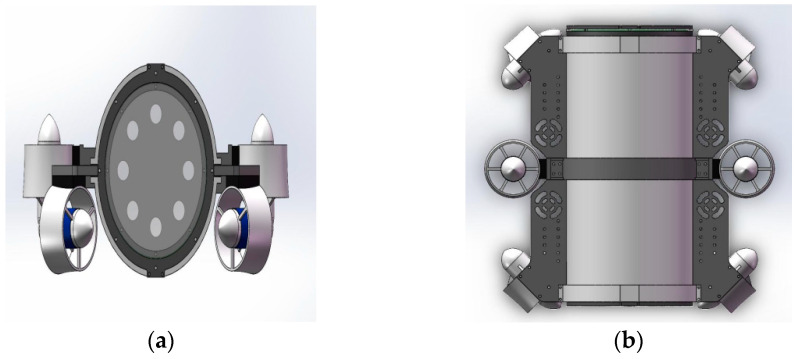
CAD model of UUV with six thrusters. (**a**) Front view. (**b**) Top view.

The process of creating the UUV model in Gazebo is as follows: firstly, SolidWorks is used to model the size, material, and shape of the underwater vehicle body, and then, the layout (installation position, connection angle, and rotation direction) of the relevant actuators is carried out. The obtained CAD model can be imported to ANSYS simulation software to calculate its mass, volume, moment of inertia, and other physical parameters. After that, the CAD model is exported into stl and dae files and based on which the xacro file is written. Finally, this UUV model can be created in Gazebo by converting the xacro file to URDF (unified robot description format) file. Figure 3 shows the UUV model used in the simulation platform, designed as a six-propeller structure, which can complete the omnidirectional movement in the horizontal plane and the up and down operation in the depth direction. In terms of power, the robot uses the PWM wave to control the power output of the propeller. The positive thrust of a single propeller is 7.5 kgf, and the reverse thrust is 5 kgf. As for size, its weight is about 9.7 kg, and its appearance size is 378 mm × 302 mm × 234 mm. In addition, the robot has been adjusted to zero buoyancy where gravity and buoyancy are equal.

**Figure 4:**
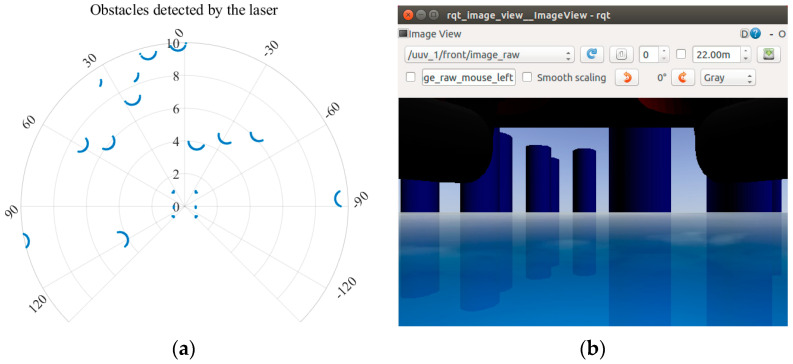
Sensor images of laser and underwater camera. (**a**) The obstacles detected by laser. (**b**) Environmental information captured by underwater camera.

To sense the environment and measure the attitude, position, and velocity of the UUV in the simulation, corresponding sensors need to be established to simulate various required signals. Open-source sensor models or self-designed models can be used as Gazebo plugins to connect to the UUV model, and the sensors (underwater camera, inertial measurement unit, laser, sonar sensor, ground truth plugin, etc.) already carried by this simulation platform are derived from the UUV simulator [28] and hector quadrotor [37] (an open-source UAV simulation platform under ROS). For example, the sensor images of the laser and underwater camera used in this work are shown in Figure 4. In addition, all sensors share a common error model:

(1) $$s=\hat{s}+n+G_{s}\;$$

(2) $$\dot{n}=-\frac{1}{\tau }+G_{b}$$

The error model is based on the first-order Gauss–Markov, and the sensing signal, s(t), at time, *t*, is given by Equations (1) and (2). Where $\hat{s}$ is the real signal, n is the current bias, *G_s_* and *G_b_* are variables describing independent zero-mean Gaussian white noise, *G_s_* is the additive noise acting directly on the measurement, and *G_b_* describes the random drift property by the time constant, *τ*.

The UUV can be considered as a rigid body model with six degrees of freedom, and its motion state can be described by the total force, F, and total torque, M, acting on it:

(3a) $${\dot{P}}^{w}=v^{w}\;$$

(3b) $${\dot{v}}^{w}=m^{-1}R_{b}^{w}F$$

(3c) $${\dot{\omega }}^{b}=J^{-1}M$$

Here, *P^w^* and ***v****^w^*are the position and velocity, respectively, of the center of gravity of the rigid body in the world inertial coordinate system, *ω^b^* is the angular rate given in the rigid body motion coordinate system, *m* and *J* are the mass and inertia of the UUV, respectively, and *R_b_^w^* is the rotation matrix which transfers the reference frame of the vector from the body coordinate system to the world coordinate system. The total force vector, *F*, includes propeller thrust, *F**_T_***, hydrodynamic forces, and hydrostatic forces; the hydrodynamic forces include lift, *F**_L_*****,** and drag, *F**_D_***, and the hydrostatic forces include gravity, *F**_G_*****,** and buoyancy, *F**_B_***. The total torque vector, *M*, is calculated by the above forces, and the specific details about hydrodynamic forces are referred to in [38], which will not be described here.

**Figure 5:**
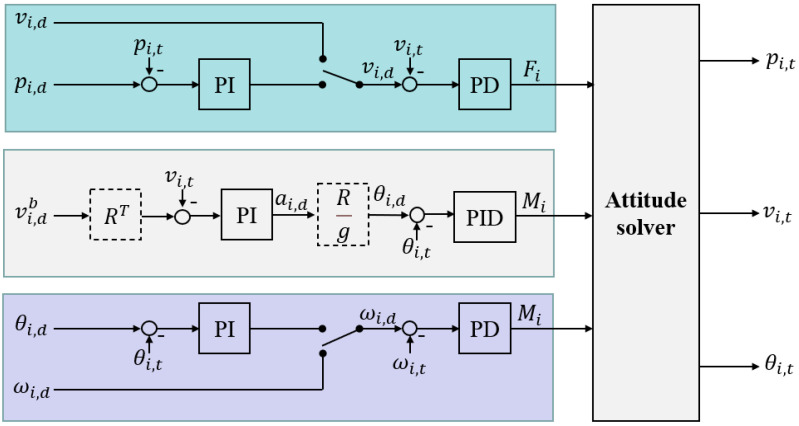
Design of controller for UUV attitude, angular velocity, and linear velocity.

We extend the control plugins in [37] based on the above dynamic model, and its control logic is shown in Figure 5. There are three control loops that control the linear velocity, attitude, and angular velocity of the UUV, respectively. The control quantity is converted into the force or torque of the corresponding axis through the cascaded PI, PD, or PID controllers, and finally output to the attitude solver. The variables, *p*, *v*, *a*, *θ*, and *ω*, respectively, represent position, linear velocity, acceleration, angle, and angular velocity, while the subscript i can be one of the three coordinate axes, X, Y, and Z; the subscript d is the expected quantity, meanwhile, the subscript t is the real quantity.

## 3. Co–Simulation Template Based on Matlab and ROS

In this section, to build a programming interface for Matlab, a co−simulation template based on Matlab and ROS is established, and simulation cases, such as path planning and task scheduling, are demonstrated based on it.

**Figure 6:**
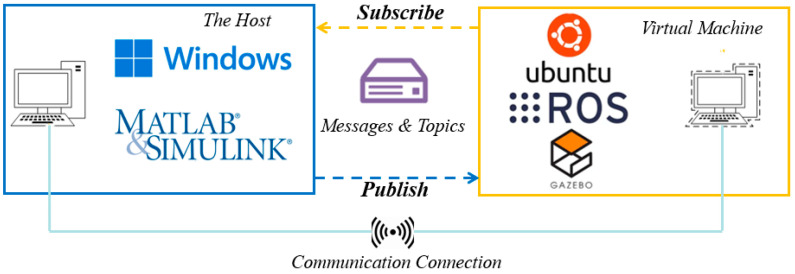
The schematic diagram of co-simulation based on Matlab and ROS.

**Figure 7:**
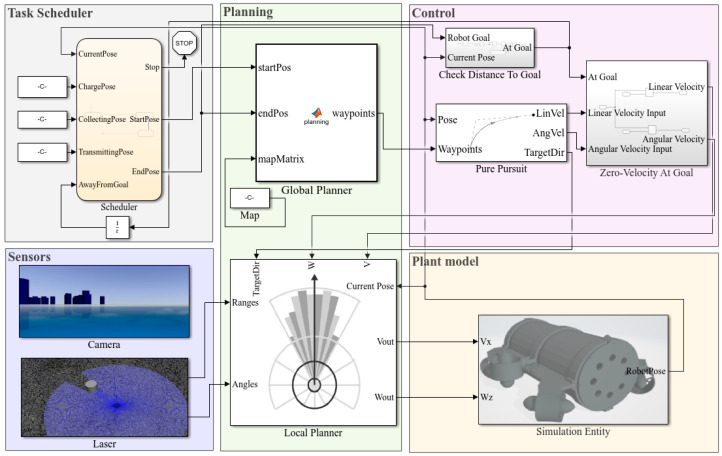
The comprehensive high-level planning template in Matlab/Simulink.

The schematic diagram of the co-simulation based on Matlab and ROS is shown in Figure 6, with Matlab/Simulink as the control terminal and ROS/Gazebo as the controlled terminal. The controlled terminal handles the physics of the rigid body (UUV), while the attached plugins are responsible for sensor reading and motion controlling. After establishing communication, the control terminal can perform high-level planning (see Figure 7 for details) based on images and sensor data acquired from virtual sensors, which is done by subscribing to the corresponding topics and publishing commands. In principle, the control terminal and the controlled terminal need to be distributed on different devices. However, a virtual machine can be created as another device, and this approach is adopted in this study. The Matlab version of the control terminal is R2021b under the Windows operating system, and the ROS version of the controlled is Kinetic under the Ubuntu 16.04 operating system.

In Figure 7, a comprehensive, high-level planning template is created in which the task scheduler based on Stateflow is responsible for transforming complex tasks into a series of events triggered by temporal conditions or external input signals. Then, the plan module is responsible for planning a collision-free path according to the starting and ending positions output by the task scheduler. The plan module contains the global planner and the local planner, which can be used separately or jointly according to the requirements, and it is worth mentioning that the binary raster map of the simulation scene should be received through the map before planning. The global planner uses the A-star algorithm to output a set of waypoints for the control module, and the control module uses the pure pursuit algorithm to calculate the expected velocity and angular velocity and publish them to the velocity topic in the plant model. The local planner mainly uses the vector field histogram (VFH) algorithm and adjusts the velocity by combining the expected linear velocity and angular velocity calculated by the control module with the scanning information of the laser in the sensor module to avoid the obstacles in the environment while executing the path following task. The plant model can also subscribe to the position and attitude information of the UUV in ROS/Gazebo, and the camera and laser included in the sensor module support the visual display. In addition, the template is modular, allowing users to design autonomous scheduling schemes, planning algorithms, etc. for testing and performance evaluation.

**Figure 8:**
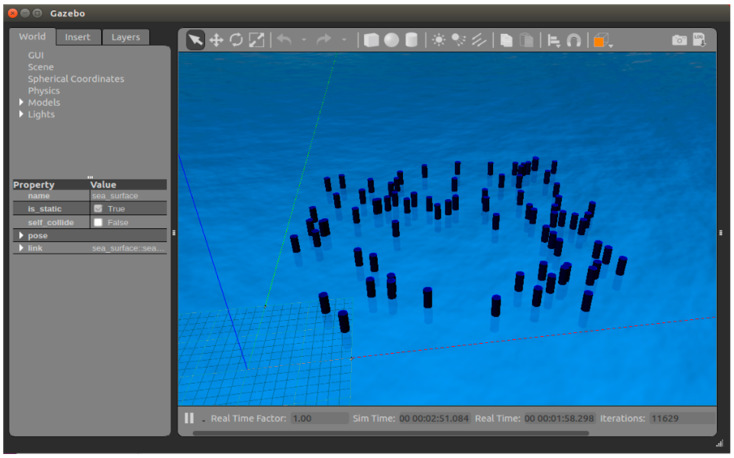
The simulation scenario in ROS/Gazebo.

Subsequently, we simulated the path-planning task, and the simulation scenario in Figure 8 is a 50 m × 50 m water area with obstacles; each obstacle is higher than the UUV and on the same plane. The specific task is to conduct global path planning by the A-star algorithm and local obstacle avoidance by the VFH algorithm to control the UUV moving from position (0,0) to position (50,50).

**Figure 9:**
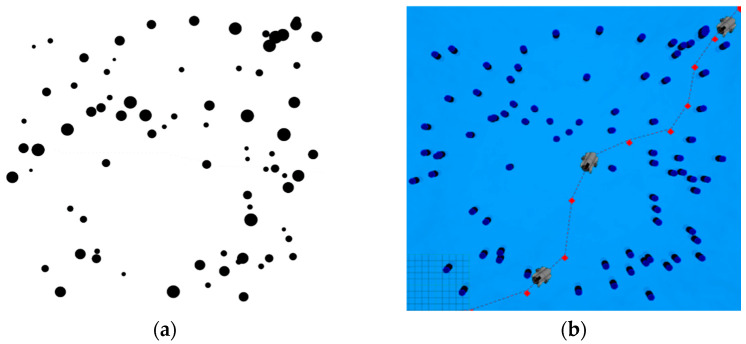
The simulation results of path-planning task. (**a**) Convert Gazebo world file to PGM map. (**b**) The trajectory of the UUV in the simulation platform.

Before planning in Matlab\Simulink, a binary raster map should be imported. The open-source function package, pgm_map_creator [39], is used to scan the Gazebo world file (Figure 8) into the portable gray map file format (PGM) map (Figure 9a); then, the obtained PGM map is rasterized and imported into the plan module. In addition, the planned trajectory is shown in Figure 9b.

**Figure 10:**
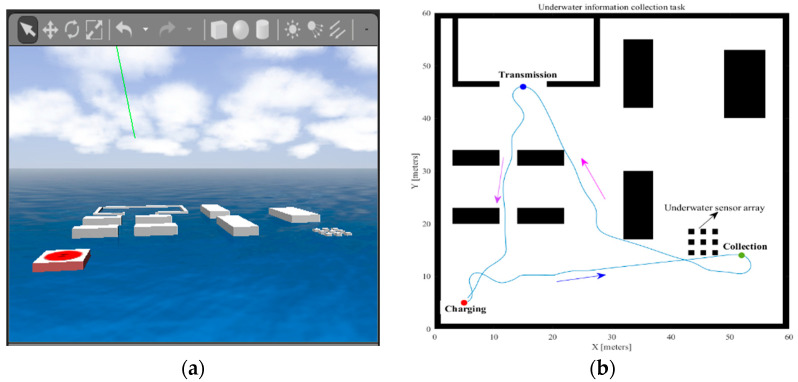
The simulation results of scheduling task. (**a**) Simulation scenario for task scheduling. (**b**) The trajectory of the UUV in the simulation platform.

The above simulation case is mainly realized based on the plan module. To verify the performance of the task scheduler, an underwater information collection task is constructed. This example demonstrates how to make the UUV perform an information gathering task on a given map. The UUV is expected to visit three locations on the map: the charging station at point (5,5), the acquisition station at point (53,13), and the transmission station at point (15,46); meanwhile, the order of access to these locations is determined by the scheduler, which provides the UUV with a target point at each stage for navigation. The simulation results are presented in Figure 10; the UUV completes the task and returns to the start point avoiding obstacles under the control of the scheduler.

## 4. UUV Formation Control Simulation Case and Underwater Experimental Verification

In this section, a formation control scheme based on the communication-constrained potential field and the virtual structure method [40] is proposed for target hunting, and the reliability of the scheme is verified in the simulation platform through the Python programming interface, and further underwater experiments are carried out.

### 4.1. Formation Control Scheme Based on Potential Field Model and Virtual Structure

The core of the formation control is to comprehensively consider the surrounding environment and maintain real-time communication with nearby robots to make the robot cluster move toward the targets in the given formation. As an earlier proposed local path-planning algorithm, the artificial potential field method has been widely used in robot formation control because of its simple mathematical model and convenient real-time control. The basic idea is to abstract the motion of the robot in the real environment into the involuntary motion under the virtual potential field. For example, the gravitational field generated by the target has a gravitational effect on the robot, and the repulsive field generated by the obstacles has a repulsive effect on the robot. Then, the force at any point in the environment is the superposition of all potential fields. The robot starts from the initial position and reaches the target point along the direction of the fastest descent of the potential field. In this study, the artificial potential field is improved, and the communication constrained potential field is added, and the specific model is as follows:

*A.*  *Gravitational potential field*

The gravitational field is proportional to the quadratic of the distance between the robot and the destination, and the gravitational field can be defined as [41]:

(4) $$P_{a}(r)=\frac{1}{2}\varepsilon_{a}r^{2}$$

(5) $${\vec{F}}_{a}=-\nabla G_{a}(r)=(-\varepsilon_{a}x,-\varepsilon_{a}y)$$

where, *P**_a_*** is the gravitational potential, *r* is the distance between the robot and the destination, *ε_a_* is the gravitational constant, and *F**_a_*** is the gravitational force on the robot at point (*x*,*y*).

*B.*  *Repulsion potential field*

While moving toward the target, the robot should also avoid obstacles and nearby robots, which are the source of repulsive forces in the potential field model, and the repulsive force field can be modeled as [42,43]:

(6) $$P_{b}(r)=\left\{\begin{array}{c} \frac{1}{2}\varepsilon_{b}\times {\left(\frac{1}{r}-\frac{1}{d_{0}}\right)}^{2},r\leq d_{0}\\ \begin{array}{ccc} \begin{array}{cc} & 0 \end{array} & & , \end{array}r>d_{0} \end{array}\right\}$$

(7) $${\vec{F}}_{b}=-\nabla P_{b}(r)=\left\{\begin{array}{c} \left(\begin{array}{c} -\varepsilon_{b}x\times \left(\frac{1}{\sqrt{x^{2}+y^{2}}}-\frac{1}{d_{0}}\right)\times {\left(x^{2}+y^{2}\right)}^{\frac{-3}{2}},\\ -\varepsilon_{b}y\times \left(\frac{1}{\sqrt{x^{2}+y^{2}}}-\frac{1}{d_{0}}\right)\times {\left(x^{2}+y^{2}\right)}^{\frac{-3}{2}} \end{array}\right),x^{2}+y^{2}\leq d_{0}{}^{2}\\ \begin{array}{ccc} & \begin{array}{ccc} \begin{array}{ccc} \begin{array}{cccc} & & 0 & \end{array} & & \end{array} & & \end{array} & ,x^{2}+y^{2}>d_{0}{}^{2} \end{array} \end{array}\right\}\;$$

where *P_b_* is the repulsion potential, *r* is the distance between the robot and the destination, *ε_b_* is the repulsion constant, *d_0_* is the maximum range affected by the repulsive force, and *F_b_* is the repulsion force on the robot at point (*x*,*y*).

*C.*  *Communication-constrained potential field*

To ensure the stability of the formation system, it is necessary to make each UUV clear its role in the whole formation, which requires good communication between UUVs in a certain range. Considering the interference of the underwater environment, the UUV can only communicate with other nearby UUVs, which is the communication constraint. Therefore, a communication-constrained potential field model is constructed:

(8) $$F_{c}(r)=\left\{\begin{array}{c} \begin{array}{cc} & 0 \end{array},r<\frac{1}{2}R_{\max}\\ \varepsilon_{c}K_{l}r,r>\frac{1}{2}R_{\max}\\ \begin{array}{cc} & 1 \end{array},r>R_{\max} \end{array}\right\}$$

When the distance between UUVs is less than $\frac{1}{2}R_{\max}$, the potential field has no effect. When the distance between UUVs is greater than $\frac{1}{2}R_{\max}$,the potential field exerts a piecewise gravitational effect. *K**_l_*** is the proportionality coefficient, and ***ε_c_*** is the potential field constant, combining the gravitational potential field, repulsive potential field, and communication-constrained potential field. Thus, the resultant force on the UUV is:

(9) $$F_{\sum }=F_{a}+\sum_{i=1}^{n}F_{bi}+\sum_{i=1}^{n}F_{ci}$$

**Figure 11:**
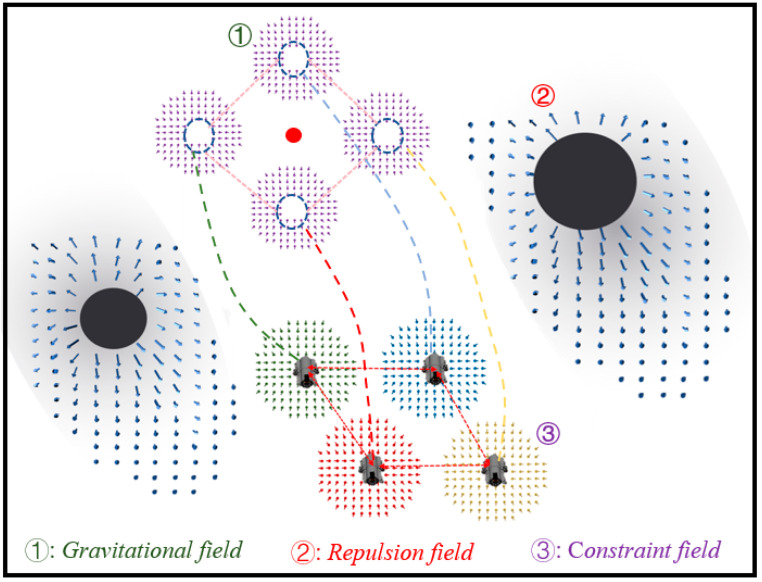
Schematic diagram of formation scheme based on improved artificial potential field and virtual structure.

The schematic diagram of the formation control scheme based on the improved artificial potential field and the virtual structure is shown in Figure 11. Firstly, the virtual structure (reference points) is set around the target, and then, the UUV moves towards the reference points under the action of gravitational potential. Under the action of the communication-constrained potential field, the UUVs will dynamically adjust the approaching speed by considering the distance difference between other UUVs and the reference points to prevent a certain UUV from moving too fast or too slow to achieve stable formation.

### 4.2. Simulation Settings and Results

**Figure 12:**
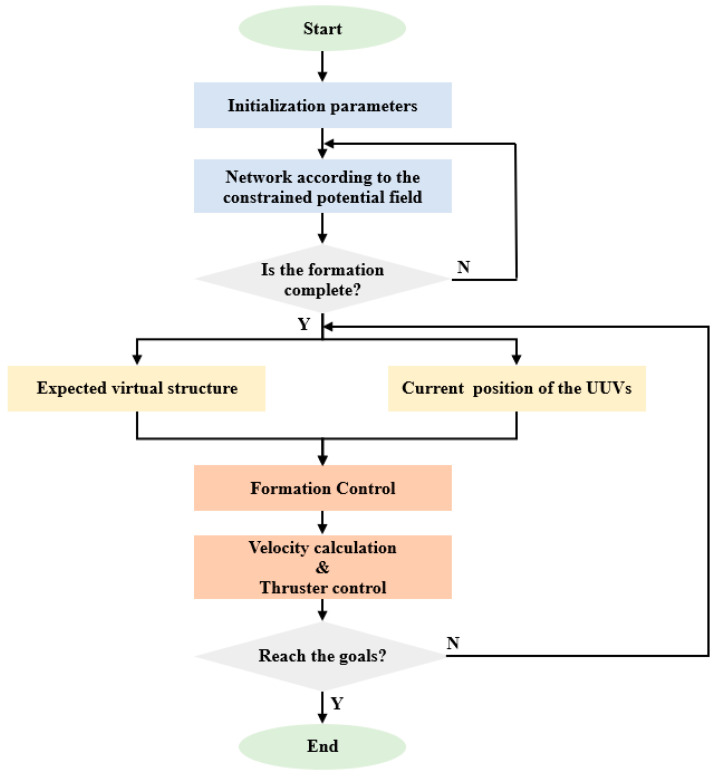
Flow chart of target-hunting simulation experiment.

The formation control scheme based on the improved artificial potential field and virtual structure is proposed in the previous section. To verify the feasibility of the scheme, the target-hunting experiment is designed and simulated in the simulation platform. The size of the experimental field is set as 10 m × 10 m, and the number of UUVs participating in the target hunting is set as 4, and the target is set at point (5,8). Gaussian noise is added to simulate the interference of the underwater environment when obtaining the real-time position of the UUV; the details are in Equation (10). In the following simulation experiments, UUVs are arranged as “line” and “diamond” formations to hunt the target respectively, and the simulation process is shown in Figure 12.

(10) $$\left\{ \begin{array}{l} L(x)=L{\left(x\right)}_{actual}+ae^{\frac{\mu_{x}{}^{2}}{2\times c^{2}}}\\ L(y)=L{\left(y\right)}_{actual}+ae^{\frac{\mu_{y}{}^{2}}{2\times c^{2}}} \end{array}\right.$$

where *L(x)* and *L(y)* are the positions reported to the UUV, *L(x)_actual_* and *L(y)_actual_* are the exact positions of the UUV, *a* and *c* are the parameters of the Gaussian noise, which can adjust the noise size and error degree, while *μ_x_* and *μ_y_* are random numbers.

**Figure 13:**
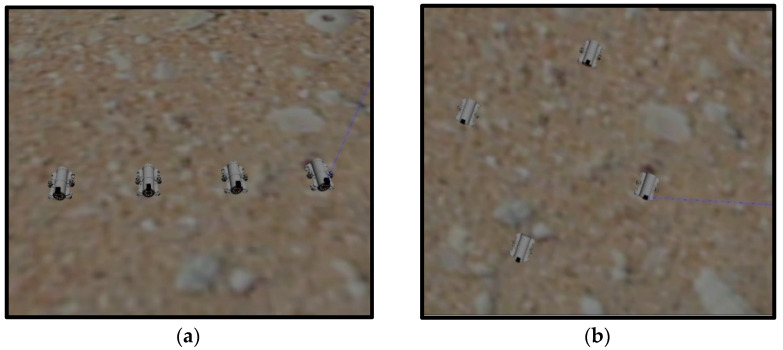

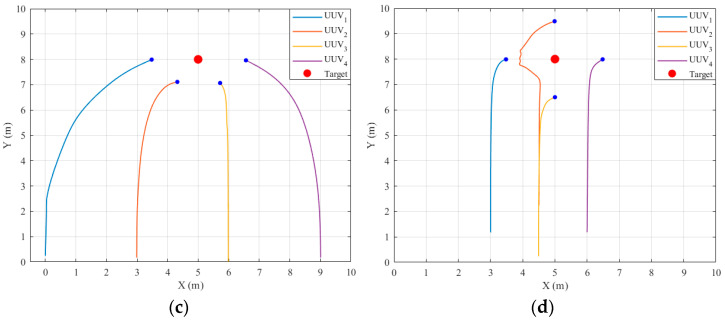
Simulation results of target hunting. (**a**,**b**) The running results of two experiments on the simulation platform. (**c**,**d**) The UUV trajectories of the two experiments.

For the “line” formation target-hunting experiment, the initial positions of the UUVs are set at points (0,0), (3,0), (6,0), and (9,0). For the “diamond” formation target-hunting experiment, the initial positions of the UUVs are set at points (4.5,2), (3,1), (6,1), and (4.5,0). The running results of the simulation platform are shown in Figure 13a,b, and the historical paths of the four UUVs are shown in Figure 13c,d, respectively. As it can be seen from Figure 13, when the UUVs approach the target, they can maintain the formation while moving towards the target, ensuring the spacing with the target and the friendly UUVs, and finally surround the target, which means that the hunting is successful.

### 4.3. Underwater Experimental Verification

**Figure 14:**
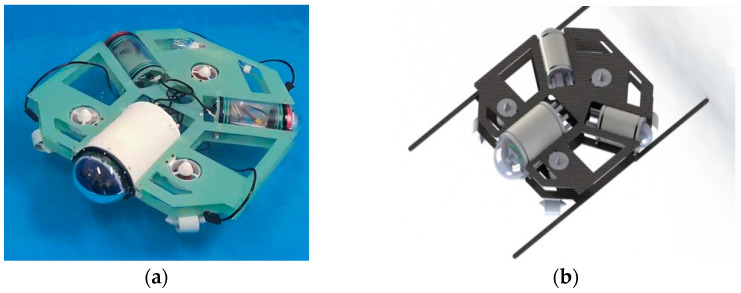
(**a**) The UUV used in the underwater experiment. (**b**) 3D model of UUV.

The above simulation results confirmed the effectiveness of our proposed formation control scheme. To further investigate the effect of the proposed formation control scheme in the real environment, the underwater experiments are carried out. The UUVs used in the experiment are designed and manufactured as in Figure 14a, and their 3D model is shown in Figure 14b.

In terms of structure, the whole UUV is powered by a 12V lithium battery, and the control cabin includes a main cabin and two side cabins, all which are round cabins. A Raspberry PI 4B microcontroller is installed in the main cabin to realize UUV environment awareness and algorithm planning, and an STM32 development board is also configured as its lower machine to control the thrusters; batteries and charging modules are placed in the side cabins.

**Figure 15:**
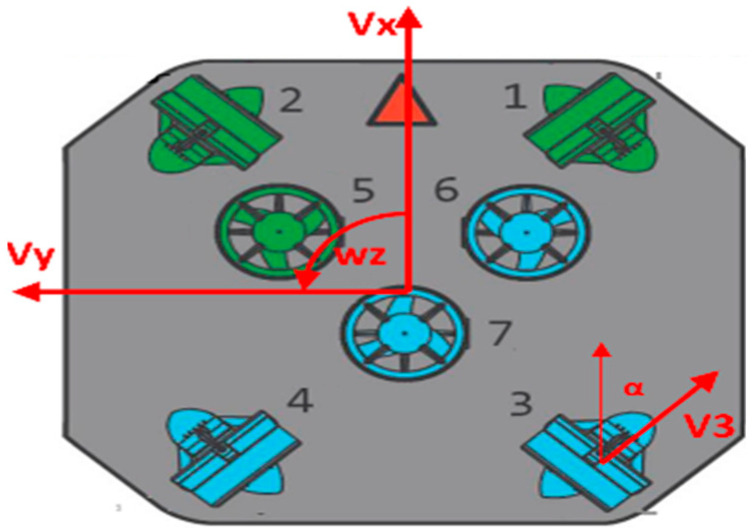
The distribution of the thrusters.

In terms of power, the mobile carrier of the UUV is mainly composed of seven thrusters, two upper and lower support plates, and four electrode plates. The distribution of the thrusters is shown in Figure 15. The thrusters numbered 1–4 are used to control the movement of the UUV on the horizontal plane, and the adjacent two thrusters are arranged at 90°. The remaining three propellers are used for the UUV ascending and descending motion, which are arranged in an angle between the hull, and the coordinated operation of the seven propellers can help the UUV achieve omnidirectional motion. Firstly, the control module will calculate the movement speed (V_x_,V_y_,W_z_) required by the UUV through PID and then adjust the speed of each thruster according to the power distribution scheme. Taking the thruster 3 in the horizontal direction as an example, when the thruster rotates to the forward direction, it will provide the UUV with a velocity of *V*_3_ at an angle, *α*, to the Y-axis, and the velocity can be decomposed into:

(11) $$\{\begin{array}{c} V_{x}=V_{3}\times \cos a\\ V_{y}=V_{3}\times \sin a\\ W_{z}=V_{3}\times R \end{array}$$

where *R* is the radius of the robot, and α is the angle between the thruster placement direction and the Y-axis direction of the robot. By inverting the matrix, we can inversely calculate the required speed of the thruster when the overall speed of the robot is known as follows:

(12) $$V_{3}=V_{x}+\frac{V_{y}}{\tan a}+\frac{R(\tan a+1)}{\tan a}W_{z}$$

Similarly, Equation (12) is extended to obtain the dynamic model of the four thrusters:

(13) $$\left[\begin{array}{c} V_{1}\\ V_{2}\\ V_{3}\\ V_{4} \end{array}\right]=\left[\begin{array}{ccc} 1 & \frac{1}{\tan a} & \frac{-R(\tan a+1)}{\tan a}\\ 1 & \frac{-1}{\tan a} & \frac{R(\tan a+1)}{\tan a}\\ 1 & \frac{-1}{\tan a} & \frac{-R(\tan a+1)}{\tan a}\\ 1 & \frac{1}{\tan a} & \frac{R(\tan a+1)}{\tan a} \end{array}\right]\cdot \left[\begin{array}{c} V_{x}\\ V_{y}\\ W_{z} \end{array}\right]$$

where *V*1–*V*4 are respectively the rotational speed of the four thrusters in the horizontal direction. According to Formula (13), the control execution module can decompose the overall motion speed of the UUV into the four thrusters to realize omnidirectional movement.

**Figure 16:**
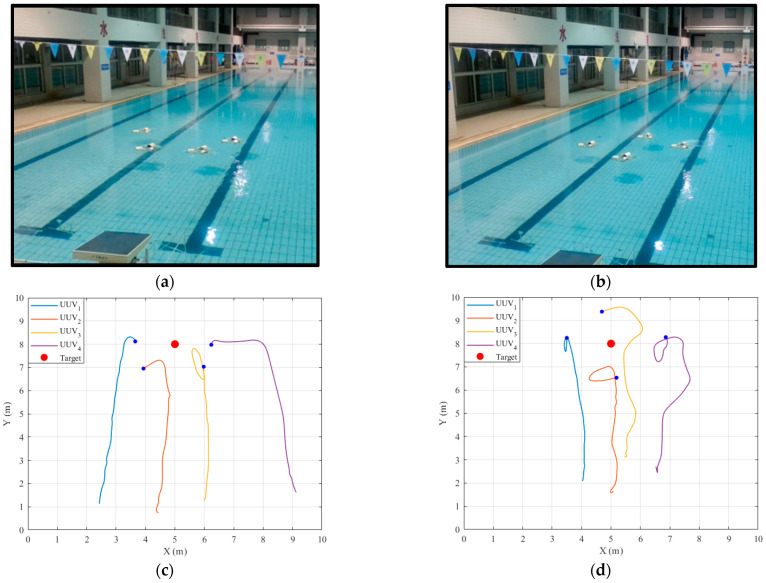
The results of the underwater experiment. (**a**,**b**) The running results of two experiments in the pool. (**c**,**d**) The UUV trajectories of the two experiments.

The settings of the underwater experiment are consistent with those of the simulation experiment in the previous section; UUVs are arranged in the formation of “linear” and “diamond” to hunt the target, respectively, and the trajectory of each UUV can be recorded in real time through the console. The experimental results are shown in Figure 16.

The simulation and underwater experiments fully verify the feasibility of the formation control scheme based on the improved artificial potential field and the virtual structure method, which can keep UUVs moving forward with the given formation under the interference of the external environment, and stably hunt the target. By comparing the simulation results (Figure 13) with the experimental results (Figure 16), the highly consistent results indicate that the fidelity of the simulation platform is reliable.

## 5. Discussion

The principle, implementation process, and verification experiments of our simulation platform are introduced above. In this section, the extensibility of the simulation platform (introducing its use in the hardware-in-loop simulation system) and comparison with other representative underwater simulation platforms will be discussed.

The hardware-in-loop (HIL) simulation is a transitional stage between software-in-loop (SIL) simulation and actual testing. It is characterized by part of the system hardware being directly placed into the simulation loop, which realizes real-time data interaction between the simulation model and the actual system and improves the fidelity of the whole model. The traditional HIL simulation systems are generally composed of the slave machine and the master machine in which the slave machine is built by the card type micro-computer, sensors, communication equipment, and other hardware. The master machine will conduct high-level planning according to the interaction information between the robot and the environment in the slave machine and issues commands to it. Hardware assembly and debugging are difficult, and the expected results are unsatisfactory. Thanks to its high integration, the simulation platform designed in this study can be loaded into the card computer to act as the slave machine without other hardware resources.

**Figure 17:**
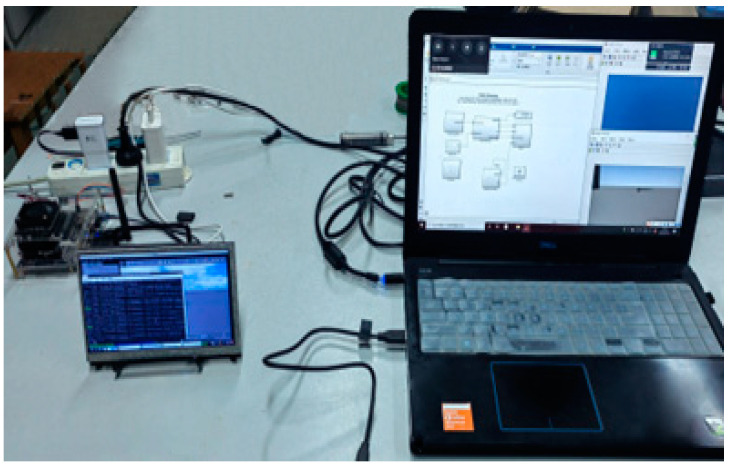
The setup of hardware-in-loop simulation system.

**Figure 18:**
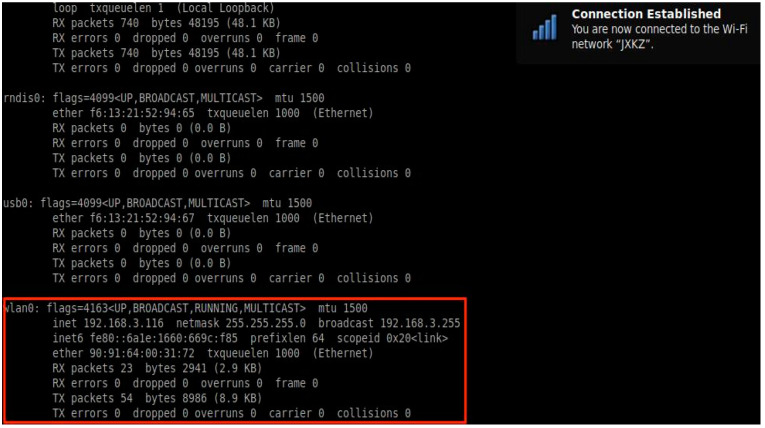
Network connection between the master and slave computers.

The setup of our hardware-in-loop simulation system is shown in Figure 17; the slave machine on the left is connected with a display screen, and the master machine is on the right. The master and slave computers communicate with each other through the wireless interface, wlan0; the network connection status is shown in Figure 18.

**Figure 19:**
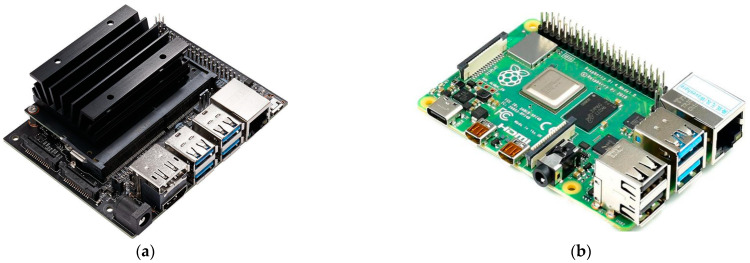
Two representative card type micro-computers: (**a**) NVIDIA Jetson Nano and (**b**) Raspberry Pi 4B.

To evaluate the environmental compatibility and operation performance of our hardware-in-loop simulation system, we successively analyzed the indicators of master CPU usage, startup time, slave memory usage, and command delay based on two representative card type micro-computers, NVIDIA Jetson Nano (Figure 19a) and Raspberry Pi 4B (Figure 19b), as shown in Table 1.

The indicators discussed in Table 1 prove that the HIL simulation system has good environmental compatibility and performance to meet the task needs, thanks to our simulation platform. To further highlight the superiority of our simulation platform, eight indicators are compared between our simulation platform and the most representative underwater simulation platforms, MVSPU [30], Stonefish [31], and UUV Simulator [28] from three aspects, as detailed in Table 2. In conclusion, our simulation platform has unique advantages in simulation accuracy, architecture design, and user experience.

## 6. Conclusions and Future Work

To guide researchers to better develop UUVs from theoretical design to simulation verification and to practical application, this study first proposed an efficient development framework in which the self-developed modular simulation platform plays an essential role. Then, the implementation process of the simulation platform is introduced in detail from the construction of the virtual environment, the UUV model import, sensor plugins, and control plugins. Subsequently, to test the interface function of the simulation platform, simulation experiments based on Matlab and Python interfaces are constructed. In the co-simulation experiment based on Matlab and ROS, we designed the high-level planning template and carried out simulation experiments, such as path planning and task scheduling. In the co-simulation experiment based on Python and ROS, the formation control scheme for target hunting based on the improved artificial potential field and virtual structure was proposed and verified. At the same time, underwater experiments were constructed; the experimental results indicate that the fidelity of our simulation platform is reliable. In addition, the application of the simulation platform in the hardware-in-loop simulation system is discussed to highlight its extensibility. Finally, the representative underwater simulation platforms are compared with our platform from different perspectives to show the unique advantages of our simulation platform.

We believe that our proposal is a useful asset for the development of robot simulation techniques, which is an interesting direction for future research because it bridges the gap between theory and experiment to compensate for the difficulties in practical testing.

Several directions exist for future work: first, our simulation platform will be extended to UAVs, unmanned vehicles, and other fields to expand its application scope; second, the optimization of the simulation calculation, such as the accurate description and modeling of the environment interference and the robot motion model, respectively; lastly, the simulation framework development and test interface will be further improved to provide simulation services for complex tasks.

## Figures and Tables

**Table 1:** Performance analysis of HIL system based on the different card type micro-computers.

Card Computer	Master CPU Usage	Startup Time	Slave Memory Usage	Command Delay
NVIDIA Jetson Nano	42%	3.2 s	37%	2 s
Raspberry Pi 4B	42%	3.6 s	59%	1.9 s

**Table 2:** Comparison of representative underwater simulation platforms.

		MVSPU [30]	Stonefish [31]	UUV Simulator [28]	Our Platform
**Environment**	Dimensions	3D	3D	3D	3D
	Precision	Low	Medium	High	High
**Features**	Distributed Architecture	No	No	No	Yes
	Programming Interface	1	1	1	Multiple
	Robotics Platform	Unity 3D	ROS	ROS and Gazebo	ROS and Gazebo
**Evaluation**	Level of Standardization	Low	Medium	Medium	High
	Difficulty of Development	Difficult	Difficult	General	Easy
	Optional Mode	SIL	SIL	SIL	SIL and HIL

## References

[1] Mohsan S.A.H., Mazinani A., Othman N.Q.H., Amjad H. (2022). Towards the internet of underwater things: A comprehensive survey. Earth Sci. Inform..

[2] Sahoo A., Dwivedy S.K., Robi P.S. (2019). Advancements in the field of autonomous underwater vehicle. Ocean Eng..

[3] Du J., Song J., Ren Y., Wang J. (2021). Convergence of broadband and broadcast/multicast in maritime information networks. Tsinghua Sci. Technol..

[4] Guan S., Wang J., Jiang C., Duan R., Ren Y., Quek T.Q.S. (2021). MagicNet: The maritime giant cellular network. IEEE Commun. Mag..

[5] Fang Z., Wang J., Ren Y., Han Z., Poor H.V., Hanzo L. (2022). Age of information in energy harvesting aided massive multiple access networks. IEEE J. Sel. Areas Commun..

[6] Fang Z., Wang J., Jiang C., Zhang Q., Ren Y. (2021). AoI-inspired collaborative information collection for AUV-assisted internet of underwater things. IEEE Internet Things J..

[7] Fang Z., Wang J., Du J., Hou X., Ren Y., Han Z. (2022). Stochastic optimization-aided energy-efficient information collection in internet of underwater things networks. IEEE Internet Things J..

[8] Yan Z., Xu D., Chen T., Zhang W., Liu Y. (2018). Leader-follower formation control of UUVs with model uncertainties, current disturbances, and unstable communication. Sensors.

[9] Hadi B., Khosravi A., Sarhadi P. (2021). A review of the path planning and formation control for multiple autonomous underwater vehicles. J. Intell. Robot. Syst..

[10] Cheng C., Sha Q., He B., Li G. (2021). Path planning and obstacle avoidance for AUV: A review. Ocean Eng..

[11] Cao X., Chen L., Guo L., Han W. (2021). AUV global security path planning based on a potential field bio-inspired neural network in underwater environment. Intell. Autom. Soft Comput..

[12] Sun Y., Ran X., Zhang G., Xu H., Wang X. (2020). AUV 3D path planning based on the improved hierarchical deep Q network. J. Mar. Sci. Eng..

[13] Lin C., Han G., Zhang T., Shah S.B.H., Peng Y. Smart underwater pollution detection based on graph-based multi-agent reinforcement learning towards AUV-based network ITS. IEEE Trans. Intell. Transp. Syst..

[14] Yuan J., Wang H., Zhang H., Lin C., Yu D., Li C. (2021). AUV obstacle avoidance planning based on deep reinforcement learning. J. Mar. Sci. Eng..

[15] He Z., Dong L., Sun C., Wang J. (2022). Asynchronous multithreading reinforcement-learning-based path planning and tracking for unmanned underwater vehicle. IEEE Trans. Syst. Man Cybern. Syst..

[16] Yan Z., Xu D., Chen T., Zhou J. (2021). Formation control of unmanned underwater vehicles using local sensing means in absence of follower position information. Int. J. Adv. Robot. Syst..

[17] Park B.S. (2015). Adaptive formation control of underactuated autonomous underwater vehicles. Ocean Eng..

[18] Yuan C., Licht S., He H. (2018). Formation learning control of multiple autonomous underwater vehicles with heterogeneous nonlinear uncertain dynamics. IEEE Trans. Cybern..

[19] Pang S.K., Li Y.H., Yi H. (2019). Joint formation control with obstacle avoidance of towfish and multiple autonomous underwater vehicles based on graph theory and the null-space-based method. Sensors.

[20] Huang H., Tang Q., Zhang G., Zhang T., Wan L., Pang Y. (2020). Multibody system-based adaptive formation scheme for multiple under-actuated AUVs. Sensors.

[21] Fernandez-Chaves D., Ruiz-Sarmiento J.R., Jaenal A., Petkov N., Gonzalez-Jimenez J. (2022). Robot@VirtualHome, an ecosystem of virtual environments and tools for realistic indoor robotic simulation. Expert Syst. Appl..

[22] Ojeda P., Monroy J., Gonzalez-Jimenez J. (2021). A simulation framework for the integration of artificial olfaction into multi-sensor mobile robots. Sensors.

[23] Rivera Z.B., De Simone M.C., Guida D. (2019). Unmanned ground vehicle modelling in gazebo/ROS-based environments. Machines.

[24] Dai X., Ke C., Quan Q., Cai K.Y. (2021). RFlySim: Automatic test platform for UAV autopilot systems with FPGA-based hardware-in-the-loop simulations. Aerosp. Sci. Technol..

[25] Xiao K., Tan S., Wang G., An X., Wang X., Wang X. XTDrone: A Customizable Multi-Rotor UAVs Simulation Platform. Proceedings of the 2020 4th International Conference on Robotics and Automation Sciences (ICRAS).

[26] Chen S., Zhou W., Yang A.S., Chen H., Li B., Wen C.Y. (2022). An end-to-end UAV simulation platform for visual SLAM and navigation. Aerospace.

[27] Liu K., Zhou X., Zhao B., Ou H., Chen B.M. An Integrated Visual System for Unmanned Aerial Vehicles Following Ground Vehicles: Simulations and Experiments. Proceedings of the 2022 IEEE 17th International Conference on Control & Automation (ICCA).

[28] Manhães M.M.M., Scherer S.A., Voss M., Douat L.R., Rauschenbach T. UUV Simulator: A Gazebo-based Package for Underwater Intervention and Multi-Robot Simulation. Proceedings of the OCEANS 2016 MTS/IEEE Monterey.

[29] Ngo A.T., Tran N.H., Ton T.P., Nguyen H., Tran T.P. Simulation of Hybrid Autonomous Underwater Vehicle Based on ROS and Gazebo. Proceedings of the 2021 International Conference on Advanced Technologies for Communications (ATC).

[30] Nie Y., Luan X., Gan W., Ou T., Song D. Design of Marine Virtual Simulation Experiment Platform Based on Unity3D. Proceedings of the Global Oceans 2020: Singapore–US Gulf Coast.

[31] Cieślak P. Stonefish: An Advanced Open-Source Simulation Tool Designed for Marine Robotics, with a ROS Interface. Proceedings of the OCEANS 2019-Marseille.

[32] Chou Y.C., Chen H.H., Wang C.C., Wang C.C., Chen W.H. A Hardware-in-the-Loop Simulation Platform for Development of AUV Control Systems. Proceedings of the 2019 IEEE Underwater Technology (UT).

[33] Kaliappan V.K., Budiyono A., Min D., Muljowidodo K., Nugroho S.A. (2012). Hardware-in-the-Loop Simulation Platform for the Design, Testing and Validation of Autonomous Control System for Unmanned Underwater Vehicle. Indian J. Geo-Mar. Sci..

[34] IHO S-57/ENC-Object and Attribute Catalogue. http://www.s-57.com/.

[35] Ramesh N.V.K., Karthik C.V.S., Yugesh J., Vani B.V., Reddy B.N.K. Analysis of Potential Regions for Maritime using QGIS Tool. Proceedings of the 2022 Second International Conference on Advances in Electrical, Computing, Communication and Sustainable Technologies (ICAECT).

[36] Zaki A., Buchori I., Sejati A.W., Liu Y. (2022). An object-based image analysis in QGIS for image classification and assessment of coastal spatial planning. Egypt. J. Remote Sens. Space Sci..

[37] Meyer J., Sendobry A., Kohlbrecher S., Klingauf U., von Stryk O. Comprehensive Simulation of Quadrotor UAVs Using ROS and Gazebo. Proceedings of the Third International Conference on Simulation, Modeling, and Programming for Autonomous Robots.

[38] Ridley P., Fontan J., Corke P. Submarine Dynamic Modelling. Proceedings of the Australian Conference on Robotics and Automation 2013.

[39] Pgm Map Creator. https://github.com/hyfan1116/pgm_map_creator.

[40] Zhou D., Wang Z., Schwager M. (2018). Agile coordination and assistive collision avoidance for quadrotor swarms using virtual structures. IEEE Trans. Robot..

[41] Khatib O. Real-Time Obstacle Avoidance for Manipulators and Mobile Robots. Proceedings of the 1985 IEEE International Conference on Robotics and Automation.

[42] Ge H., Chen G., Xu G. (2018). Multi-AUV cooperative target hunting based on improved potential field in a surface-water environment. Appl. Sci..

[43] Zhen Q., Wan L., Li Y., Jiang D. (2022). Formation control of a multi-AUVs system based on virtual structure and artificial potential field on SE(3). Ocean Eng..

